# Molecular identification of *Anaplasma marginale* in two autochthonous South American wild species revealed an identical new genotype and its phylogenetic relationship with those of bovines

**DOI:** 10.1186/s13071-016-1555-9

**Published:** 2016-05-26

**Authors:** Eliana C. Guillemi, Sofía de la Fourniere, Marcela Orozco, Jorge Peña Martinez, Elena Correa, Javier Fernandez, Ludmila Lopez Arias, Martina Paoletta, Belkis Corona, Valérie Pinarello, Silvina E. Wilkowsky, Marisa D. Farber

**Affiliations:** Instituto de Biotecnologia, Centro de Investigaciones en Ciencias Veterinarias y Agronómicas, INTA, Buenos Aires, Argentina; Laboratorio de Eco-Epidemiología, Departamento de Ecología, Genética y Evolución, Universidad de Buenos Aires - Instituto de Ecología, Genética y Evolución, CONICET, Buenos Aires, Argentina; The Conservation Land Trust, Corrientes, Argentina; Reserva Experimental Horco Molle, Facultad de Ciencias Naturales, Universidad Nacional de Tucumán, Tucumán, Argentina; National Center for Animal and Plant Health, Apartado 10, postal address 32700 San José de las Lajas, Mayabeque Cuba; CIRAD UMR 15/UMR CIRAD-INRA 1309 “contrôle des maladies animales exotiques et émergentes”, Domaine Duclos, Prise d’eau, 97170 Petit Bourg, Guadeloupe

**Keywords:** *Anaplasma marginale*, Wild species, Taruca, Giant anteater

## Abstract

**Background:**

*Anaplasma marginale* is a well-known cattle pathogen of tropical and subtropical world regions. Even though, this obligate intracellular bacterium has been reported in other host species different than bovine, it has never been documented in *Myrmecophaga tridactyla* (giant anteater) or *Hippocamelus antisense* (taruca), which are two native endangered species.

**Methods:**

Samples from two sick wild animals: a *Myrmecophaga tridactyla* (blood) and a *Hippocamelus antisense* (blood and serum) were studied for the presence of *A. marginale* DNA through *msp5* gene fragment amplification. Further characterization was done through MSP1a tandem repeats analysis and MLST scheme and the genetic relationship among previously characterized *A. marginale* sequences were studied by applying, *eBURST* algorithm and AMOVA analysis.

**Results:**

*Anaplasma marginale* DNA was identified in the *Myrmecophaga tridactyla* and *Hippocamelus antisense* samples. Through molecular markers, we identified an identical genotype in both animals that was not previously reported in bovine host. The analysis through *eBURST* and AMOVA revealed no differentiation between the taruca/anteater isolate and the bovine group.

**Conclusions:**

In the present publication we report the identification of *A. marginale* DNA in a novel ruminant (*Hippocamelus antisense*) and non-ruminant (*Myrmecophaga tridactyla*) host species. Genotyping analysis of isolates demonstrated the close relatedness of the new isolate with the circulation population of *A. marginale* in livestock. Further analysis is needed to understand whether these two hosts contribute to the anaplasmosis epidemiology.

## Background

*Anaplasma marginale* is an obligate intracellular pathogen from the phylum Proteobacteria, class alpha Proteobacteria, order Rickettsiales, and family Anaplasmataceae. *Anaplasma marginale* is known as an intraerythrocytic pathogen that causes moderate to severe hemolytic anemia, jaundice and hemoglobinuria without hemoglobinemia in cattle [1], although, other host species different than bovine were reported to be infected by this bacteria. In previous publications, *Mazama gouazoubira, Blastocerus dichotomus*, *Syncerus caffer*, *Bubalus bubalis*, *Oryx gazella*, *Camelus dromedarius*, *Kobus vardonii* and *Aepyceros melampus* have been described as *A. marginale* hosts [2–7]. The economic losses generated by anaplasmosis are not only associated with morbidity and mortality in cattle, but also with a lower weight gain rate, lower milk production, abortions and treatment costs. The identification of new hosts, the study of the genotypes associated to wild species and their interactions with genotypes frequently found in livestock could improve the complete understanding of the eco-epidemiology of anaplasmosis, and will be beneficial for surveillance and disease control. In the present study, we describe the identification of *A. marginale* DNA in two wild species (*Myrmecophaga tridactyla* and *Hippocamelus antisensis*) where this pathogen has not been previously reported. After molecular diagnosis of *A. marginale* by amplifying a fragment of the *msp5* gene, both isolates were characterized applying two different molecular markers. Through MSP1a tandem repeats analysis and the *A. marginale* MLST scheme we were able to characterize the genotypes and study their geographical distribution. Results suggest that *A. marginale* could be circulating in *Myrmecophaga tridactyla* and *Hippocamelus antisensis* and that there seems to be directionality in the transmission of certain genotypes from cattle to these wild species.

## Methods

### Case reports

#### Case 1

**Fig. 1:**
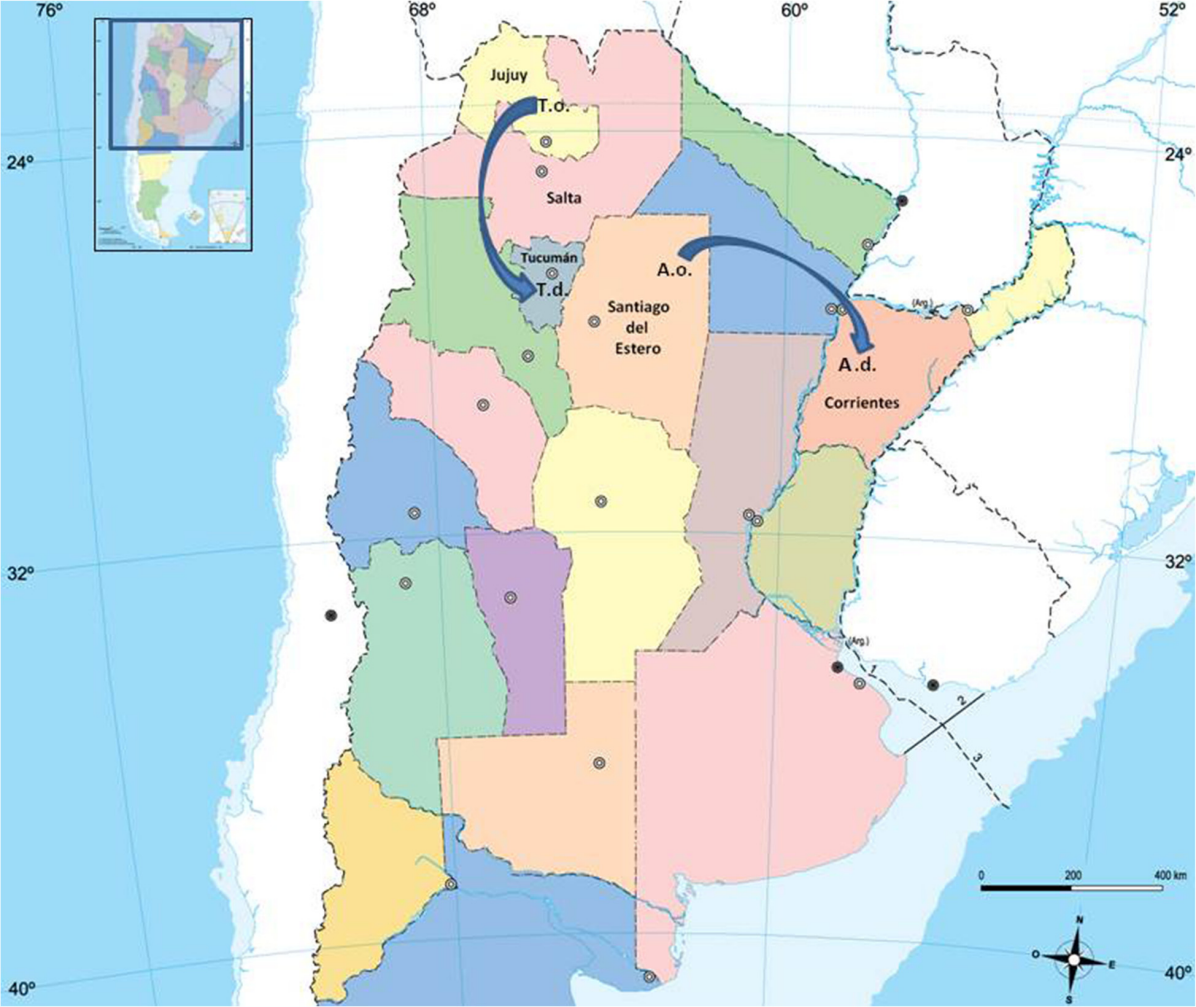
Map representing the north of Argentina and the taruca and the giant anteater movements. T.o: Taruca origin, T.d: Taruca destination, A.o: anteater origin and A.d: anteater destination

**Fig. 2:**
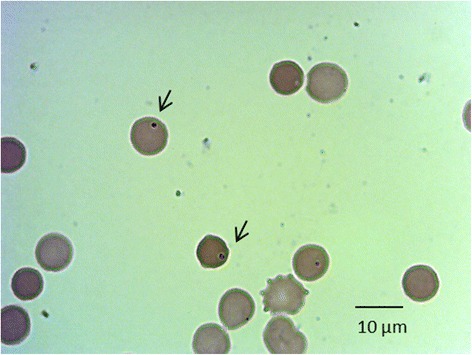
Blood smear from *Myrmecophaga tridactyla.* The arrows point out spherical inclusions suggestive of *A. marginale.* May Grunwald-Giemsa-Giemsa, 100× oil immersion

In May 2013, as part of a reintroduction program carried out in Corrientes province, Argentina, a 20 days old female of giant anteater (*Myrmecophaga tridactyla*) (Xenarthra, Myrmecophagidae) had been released from illegal trafficking and transferred from Santiago del Estero province (Argentina) to the rescue center in Corrientes province (Argentina) (Fig. 1). One year later, a blood smear examination showed intraerythrocytic structures suggestive of *A. marginale* (Fig. 2). Finally, on 28 October 2014, the anteater died and venous blood samples were remitted to our laboratory for *A. marginale* molecular diagnosis.

#### Case 2

**Fig. 3:**
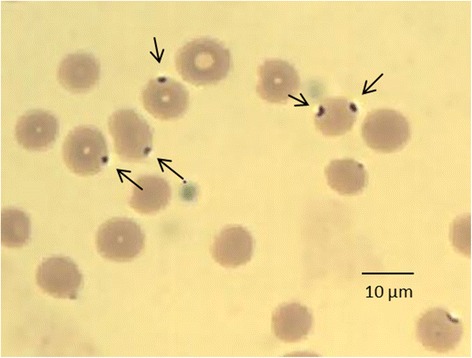
Blood smear from *Hippocamelus antisensis.* The arrows point out spherical inclusions suggestive of *A. marginale.* May Grunwald-Giemsa-Giemsa, 100× oil immersion

In October 2012, a nine month old north Andean deer “taruca” (*Hippocamelus antisensis*) (Cetartiodactyla, Cervidae) arrived to the “Reserva Experimental de Flora y Fauna de Horco Molle” in Tucuman province (Argentina) as a result of a wildlife rescue procedure. The original location of the cervid was a forest area in Jujuy province (Argentina) (Fig. 1). The cervid underwent a pre-surgical analysis and a significant low PCV value was detected (17 %) in the absence of bleeding history or bloody feces. Intraerythrocytic structures suggestive of *A. marginale* were observed after blood smear examination (Fig. 3) and blood samples were remitted to our laboratory for *A. marginale* molecular diagnosis. Also, a frozen stored serum sample that has been taken previously (in May 2012) was sent as a background sample. In March 2014 the cervid died as a result of an infected myiasis in the head and subsequent sepsis.

In both cases, samples were collected for routine diagnostic purposes following institutional guidelines. Capture and transit permits (Reference numbers: 1140 and 000336) were obtained from the provincial government through Natural Resources Agency of Corrientes and Tucuman, respectively.

### Samples and genomic DNA isolation

Blood samples from the taruca and the giant anteater (one per animal) and a serum sample from the taruca were received and analyzed in our laboratory for *A. marginale* identification. The genomic DNA extraction from blood and serum samples was performed by phenol/chloroform method and a standard ethanol precipitation [8].

### PCR assays for *A. marginale*

The first assay was based on the amplification of a fragment from a single copy gene that encodes the outer major surface protein MSP5 from *A. marginale*. Reaction and amplification conditions were carried out as described by Torioni de Echaide et al. [9]. For further isolate characterization two molecular marker schemes were employed. First, *msp1α* gene was amplified and sequenced for identifying the number and type of tandem repeats in the 5’region of the gene, according to the protocol described by [10]. Secondly, a multilocus sequence type (MLST) scheme based on the allelic polymorphism of seven *A. marginale* housekeeping genes (*dnaA, ftsZ, lipA, groEl, recA, secY* and *sucB*) was applied [11]. The nucleotide sequence of the primers employed and the resulting amplicon size are shown in Table 1.

**Table 1:** Primers employed for *A. marginale* identification and characterization

Gene	Product	Primer sequence	Amplicon size (bp)	Reference
*msp5*	major surface protein MSP5	F: 5' GCATAGCCTCCCCCTCTTTC 3'	548	[9]
		R: 5' TCCTCGCCTTGCCCCTCAGA 3'		
		F: 5' TACACGTGCCCTACCGACTTA 3'	345	
		R: 5' TCCTCGCCTTGCCCCTCAGA 3'		
*msp1α*	major surface protein MSP1a	F: 5'GCATTACAACGCAACGCTTGAG 3'	84–87 bp tandem repeats	[10]
		R: 5'GCTTTACGCCGCCGCCTGCGCC 3'		
*dnaA*	Chromosomal replication initiation protein	F: 5' GTTCATAAGCGGGAAGGACA 3'	512	[11]
		R: 5' CTTGTCTCGGTCTGGCTAGG 3'		
*ftsZ*	Cell division protein FtsZ	F: 5' CCTGACCACCAATCCGTATC 3'	575	
		R: 5' CCCGTATGAAGCACCGTATC 3'		
*groEl*	Chaperonin GroEL	F: 5' AGCATAAAGCCCGAGGAACCTT 3'	699	
		R: 5' GCCGAGCATGTCCTTCCTTCTG 3'		
*lipA*	Lipoyl synthase	F: 5' TGTGGATAGGGACGACCTTC 3'	538	
		R: 5' AAAGTCATCCTCAGCGTGGT 3'		
*recA*	Recombinase A	F: 5' GGGCGGTAACTGTGCTTTTA 3'	579	
		R: 5' ACGCCCATGTCGACTATCTC 3'		
*secY*	Preprotein translocase subunit SecY	F: 5' TTCACGCTGCTAGCCCTAAT 3'	501	
		R: 5' TACGAGGGAAATGCCGTTAC 3'		
*sucB*	Dihydrolipoamide acetyltransferase component	F: 5' GAGATAGCATCTCCGGTTGC 3'	808	
		R: 5' CTCCCCTGGCCTTTTTACTC 3'		

### Molecular markers analysis

Multiple alignments of the *msp1α* sequences obtained for the taruca and the anteater isolates were performed using the Clustal W2 software (EMBL-EBI, Wellcome Trust Genome Campus, Hinxton, Cambridgeshire, UK). After the alignment, nucleotide sequences were translated to amino acid sequences in order to identify the tandem repeats. The repeat patterns were compared to those previously published [12, 13].

For automate MLST-DNA sequence editing and analysis, a custom-designed bioinformatic “Galaxy MLST-Pipeline” has been employed (http://bioinformatica.inta.gov.ar/galaxy/) [11]. The information obtained from this pipeline was then used for inferring relatedness between allelic profiles through the eBURST algorithm [14]. The sequence type (ST) from 74 *A. marginale* strains was retrieved from the MLST-Pipeline database (MLST-DB) (http://bioinformatica.inta.gov.ar/mlst/) for the study of the genotype diversity and distribution of the new isolates. This database includes information relating to strains from cattle of diverse geographic origins (USA, Puerto Rico, Italy, South Africa, Mexico, Colombia, Cuba, Brazil, Argentina and Uruguay). Applying the PHYLOViZ program [15], a triple locus variant (TLV) criteria was employed for the clonal complexes (CC) construction, meaning that those related genotypes that differ in up to three genes from the founder genotype will be arranged in the same CC. Also the full MLST was run; this option links all STs through the construction of a Minimum Spanning Tree and specifying the number of alleles that are different between each pair of STs. Also, the Arlequin 3.1 program [16] was employed to assess how much of the total genetic variation (measured by ST) was partitioned between the isolates through AMOVA [17]).

## Results

### Diagnosis and genotyping of *A. marginale*

**Fig. 4:**
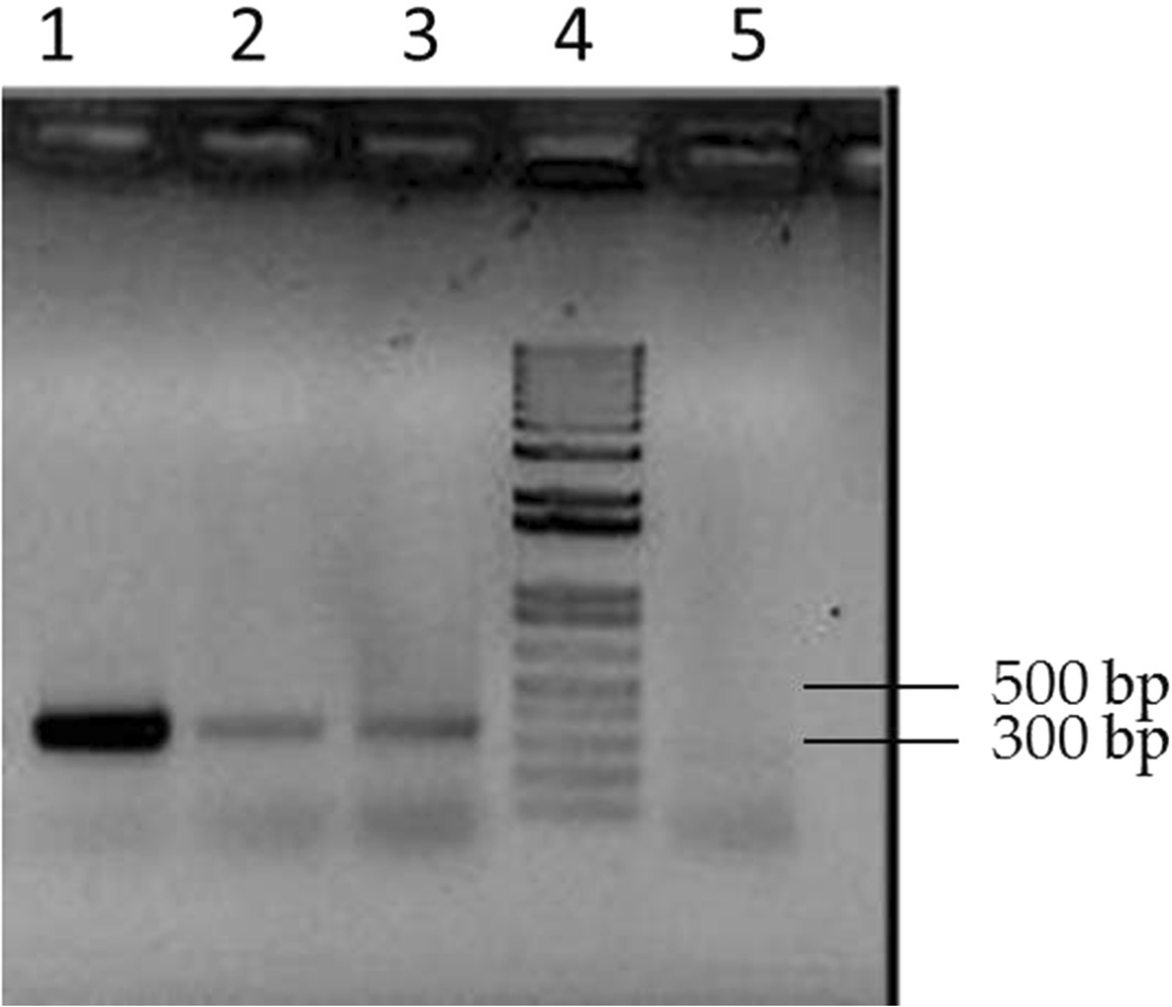
Agarose gel electrophoresis of *msp5* gene amplification. 1 % agarose. Lane 1: positive control; Lane 2: anteater sample; Lane 3: taruca sample; Lane 4: Molecular marker (1 Kb plus); Lane 5: negative control

Samples from the taruca (blood and serum) and the giant anteater (blood), tested for *A. marginale* with the *msp5* specific assay, were PCR-positive (Fig. 4). After confirming the presence of *A. marginale* DNA, both isolates were characterized from blood samples by the *msp1α* marker and the MLST scheme. The MSP1a tandem repeat profile of the taruca and the anteater displayed the same genotype. The MSP1a profile consisted on two tandem repeats (repeat 13 and repeat 27) that were previously found in other genotypes, but were not previously reported in this combination, thus resulting in a new MSP1a genotype (Table 2).

**Table 2:** Amino acid sequence for MSP1a tandem repeats found for the taruca and the giant anteater *A. marginale* isolates

Repeat	Encoded sequence	Number of repeats found
13	TDSSSASGQQQESSVLSQSDQASTSSQLG	1
27	ADSSSASGQQQESSVLSQSDQASTSSQLG	1

For the MLST scheme, both isolates were assigned the same ST. Each one of the seven alleles was already registered in the MLST-DB, though the combination of alleles retrieved a new ST that resulted unique for the taruca and the anteater *A. marginale* isolate (Table 3). Sequences from *msp1α* and from the seven MLST genes were deposited in GenBank (submission ID: 1892187).

**Table 3:** Alleles found for each gene and the resulting MLST scheme ST

ST	*dnaA*	*ftsZ*	*groEl*	*lipA*	*recA*	*secY*	*sucB*
69	3	3	2	2	13	4	4

### Genetic relationship among isolates

**Fig. 5:**
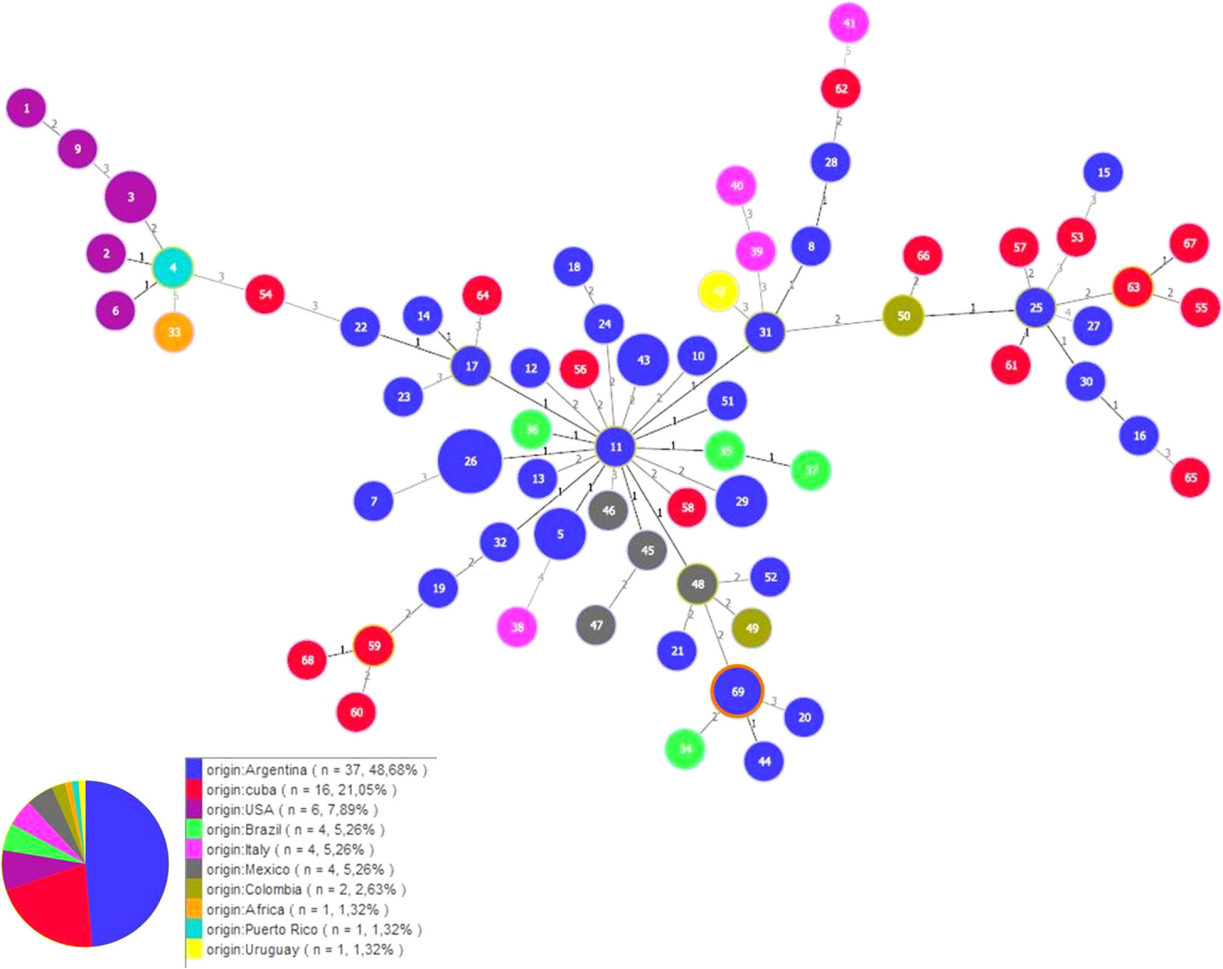
eBURST representation of genetic relationship among isolates. ST 69 is highlighted with an orange circle

Applying eBURST algorithm with a TLV criteria, a unique CC was found. USA isolates were arranged together around ST 4 (Puerto Rico). The rest of the CC was comprised of STs from diverse Latin-American countries (Argentina, Brazil, Uruguay, Mexico, Cuba and Colombia) and two STs from Italia (STs 39 and 40), all of them arranged around the ST 11 (Argentina). In particular, ST 69, which corresponds to the taruca and the giant anteater isolates, was established as a link between one ST from Brazil (ST 34) and two STs from northwest Argentina (STs 20 and 44), specifically from Salta, a neighboring province of Jujuy (Figs. 1 and 2). The South African ST (ST 33), two STs from Italy (STs 38 and 41) and one from Argentina (ST 27) remained as singletons (not integrated to the CC). The full MLST run showed the two main parts of the CC linked through ST 54 from Cuba (Fig. 5). Thus, we considered the genotypes that differ in up to two genes from the founder genotype (double locus variant criteria) to come up with a CC comprised of STs from Latin-American countries and the taruca/anteater isolates (STs linked by 1 and 2, Fig. 5). We compute the pairwise population comparisons of genetic differentiation between populations (F_*ST*_), and no differentiation was shown between the taruca/anteater isolate and the bovine group (F_*ST*_ = -0.04699), revealing that most of the genetic diversity was observed among individuals within populations.

## Discussion

The comprehensive study of the anaplasmosis epidemiology needs the identification of all the involved factors, including susceptible, carrier and potential reservoir hosts. Herein, the evidence of *A. marginale* presence in the taruca and the giant anteater samples was supported not only by smear examination but remarkably by the PCR amplification of nine different gene sequences: one gene fragment currently used for diagnosis (*msp5*) and eight for molecular typing (*msp1a* and the seven neutral fragments from MLST scheme). Notably, the genotyping data revealed the presence of the same *A. marginale* isolate in both wild species.

Relevant information that arises from the results obtained here is the presence of *A. marginale* DNA in the taruca serum sample. Although serum samples are not ideally suited for genomic DNA extraction, especially from intraerythrocytic agents, we could manage to obtain a sufficient amount of *A. marginale* DNA for PCR detection. This fact probably has to do with the presence of the dense (infective) forms of *A. marginale,* which are able to survive outside the erythrocyte before parasitizing another cell [1]. The presence of *A. marginale* DNA in the taruca serum is an important fact since this sample was obtained two years before the blood sample, when the cervid was in its original location in Jujuy province, indicating that the taruca was already infected when moved to Horco Molle in Tucuman province.

Even though *A. marginale* is the most prevalent tick-borne disease in livestock worldwide and studies of its impact are mainly focused in domestic cattle. [1], this bacteria has also been detected in blood from other wild mammalian ruminant species such as *Mazama gouazoubira*, *Blastocerus dichotomus*, *Syncerus caffer*, *Bubalus bubalis*, *Oryx gazella*, *Camelus dromedarius*, *Kobus vardonii* and *Aepyceros melampus* [2–7, 18]. To date, there are no reports describing the presence of *A. marginale* in a taruca (*Hippocamelus antisensis*) and/or giant anteater (*Myrmecophaga tridactyla*), two native endangered species. Moreover, the finding in the giant anteater is the first demonstration of *A. marginale* in a non-ruminant species. Other *Anaplasma* species, *A. phagocytophilum*, has been found in a great diversity of hosts including human. This species has been considered a generalist pathogen with highly adaptive strategies that enables this bacterium to infect a wide range of hosts [18]. Recently, employing the *groEL* gene as a molecular marker Jahfari et al. [19] could discriminate four different *A. phagocytophilum* ecotypes with significantly different host ranges and zoonotic potential; these findings suggest that certain genotypes may be adapted to specific host species. It is likely that, this ‘wide host adaptation’ ability may be a common feature to other *Anaplasma* species, and this could explain why *A. marginale* is found in other host species different than cattle.

In Argentina the enzootic area for *A. marginale*, extends from the northern boundary of the country to the parallel 33 ° S. The main vector implicated in anaplasmosis transmission is *Rhipicephalus microplus*, an Ixodidae tick distributed through the north of the country. While not in anteater, there are reports of *R. microplus* parasitizing various cervid species [20, 21]. Also, other tick genus has been implicated as *A. marginale* vectors [22]. In this sense, *Amblyomma cajennense*, could be a potential *A. marginale* vector [23] and was previously reported parasitizing cattle [21, 24], giant anteater [24–26] and cervids [20, 21], highlighting the importance of following up its role as a source of infection of *A. marginale* for domestic and wild species. Moreover, *A. cajennense* is well distributed through Jujuy, Tucuman and Santiago del Estero province in Argentina [27]. In addition to ticks, other hematophagous insects could cause mechanical transmission of *A. marginale* [1]. In fact, *Tabanus* spp. is well distributed over South America and may act as a mechanical vector between cattle and new hosts species. In the two cases reported in this study, no ticks or other hematophagous arthropod were found parasitizing the taruca nor the giant anteater neither arriving at rescue centers or during their stay there.

Additionally we provide information regarding epidemiological analysis through genotyping of both isolates and comparing them with cattle genotypes reported to date. In this sense, the ST 69 found in both wild species resulted in a new variant consisting of seven alleles previously described in cattle from Argentina and other world regions [11]. Similarly, the two tandem repeats found through the MSP1a marker (repeat 13 and 27) were previously published for *A. marginale* isolates in cattle from Argentina, Mexico, Brazil and South Africa [12], but, as they had never been found together, their combination resulted in a novel genotype.

As we have previously reported [11], there is a broad genetic diversity of *A. marginale* isolates in bovine host. Moreover, the chance of finding the same genotype in cattle samples is scarce (Fig. 5 and [12]). Identical isolates have only been detected in samples collected in the same herd at the same time point (ST 26, 29 and 43). However, taken together, the information obtained from the *msp1α* genotype, the eBURST and AMOVA analysis highlights that, although different, the newly emerged genotype found in wild species is not separated from the rest of genotypes found in bovines. This finding might suggest that transmission of the new *A. marginale* isolate from cattle to accidental hosts could have arisen by means of a better transmission vector-competent isolate or by the emergence of a favored variant with an improved fitness for wider hosts. Further evidence is needed to support this hypothesis, mainly through the characterization of other *A. marginale* isolates from a greater number of wild host species.

To our knowledge, this is the first demonstration of *A. marginale* DNA in a taruca and a giant anteater. Moreover, for the taruca, *A. marginale* was detected over time, since the bacterium DNA was present in a serum sample and in a blood sample collected two years after.

Since the anteater and the taruca sustained other pathogens simultaneously, we are unable to affirm that *A. marginale* has infected or was the cause of death of the animals. Even though DNA from *A. marginale* has been detected from both taruca and anteater blood samples, whether these two hosts species contribute to the anaplasmosis epidemiology needs further investigation.

## Conclusions

Our study demonstrated the presence of *A. marginale* DNA in two up to now non-acknowledged host species: *Hippocamelus antisense* and *Myrmecophaga tridactyla*. Moreover, this is the first report of *A. marginale* in a non-ruminant host. The sequence analysis of the molecular markers revealed that the same genotype was present in both the cervid and the anteater, and further analysis demonstrated the close relatedness of the new isolate with the circulation population of *A. marginale* in livestock. On one hand, these results could be an evidence of anaplasmosis as a threat for wild life and additionally could represent a sign of alternative mammalian reservoir for *A. marginale*. The acceptance of this hypothesis is worth an epidemiological base search and analysis.
