# Erratum to: Molecular identification of *Anaplasma marginale* in two autochthonous South American wild species revealed an identical new genotype and its phylogenetic relationship with those of bovines

**DOI:** 10.1186/s13071-016-1653-8

**Published:** 2016-06-24

**Authors:** Eliana C. Guillemi, Sofía de la Fourniere, Marcela Orozco, Jorge Peña Martinez, Elena Correa, Javier Fernandez, Ludmila Lopez Arias, Martina Paoletta, Belkis Corona, Valérie Pinarello, Silvina E. Wilkowsky, Marisa D. Farber

**Affiliations:** Instituto de Biotecnologia, Centro de Investigaciones en Ciencias Veterinarias y Agronómicas, INTA, Buenos Aires, Argentina; Laboratorio de Eco-Epidemiología, Departamento de Ecología, Genética y Evolución, Universidad de Buenos Aires - Instituto de Ecología, Genética y Evolución, CONICET, Buenos Aires, Argentina; The Conservation Land Trust, Corrientes, Argentina; Reserva Experimental Horco Molle, Facultad de Ciencias Naturales, Universidad Nacional de Tucumán, Tucumán, Argentina; National Center for Animal and Plant Health, Apartado 10, postal address 32700 San José de las Lajas, Mayabeque Cuba; CIRAD UMR 15/UMR CIRAD-INRA 1309 “contrôle des maladies animales exotiques et émergentes”, Domaine Duclos, Prise d’eau, 97170 Petit Bourg, Guadeloupe

## Erratum

After publication of our work [1], we realised that we have erroneously provided the GenBank submission ID instead of GenBank sequence accession numbers within the results section “Diagnosis and genotyping of *A. marginale*”: “Sequences from *msp1α* and from the seven MLST genes were deposited in GenBank (submission ID: 1892187)”. The corrected sentence is as follows: “Sequences from *msp1α* and from the seven MLST genes were deposited in the GenBank database under accession numbers KU879097–KU879112”.

We would like to apologise for the error and for any inconvenience this may have caused.
